# Involvement of PAR2 in platelet‐derived growth factor receptor‐α‐positive cell proliferation in the colon of diabetic mice

**DOI:** 10.14814/phy2.15099

**Published:** 2021-11-09

**Authors:** Yu‐Jia Li, Jun‐Ping Ao, Xu Huang, Hong‐Li Lu, Han‐Yue Fu, Ni‐Na Song, Wen‐Xie Xu, Jie Chen

**Affiliations:** ^1^ Department of Anatomy and Physiology Shanghai Jiao Tong University College of Basic Medical Science Shanghai China; ^2^ Department of Pediatric Surgery Xinhua Hospital Affiliated to Shanghai Jiao Tong University School of Medicine Shanghai China; ^3^ State Key Laboratory of Oncogenes and Related Genes School of Medicine Shanghai Cancer Institute Renji Hospital Shanghai Jiao Tong University Shanghai China

**Keywords:** colon, diabetic mice, PAR2, PDGFRα^+^ cells, proliferation

## Abstract

Our previous study indicated that streptozotocin (STZ)‐induced diabetes leads to colonic platelet‐derived growth factor receptor‐α‐positive (PDGFRα^+^) cell proliferation accompanied by slow colonic transit in mice; however, the mechanism of this effect is unclear. The present study used western blotting, immunohistochemistry, and quantitative PCR to investigate whether proteinase‐activated receptor 2 (PAR2) mediates PDGFRα^+^ cell proliferation. Our results showed that PDGFRα, PAR2, and Ki‐67 coexpression was increased in the diabetic colonic muscle layer. PDGFRα and PAR2 mRNA and protein expression levels were also markedly enhanced in the diabetic colonic muscle layer. Mice treated with 2‐furoyl‐LIGRLO‐amide (2‐F‐L‐a), a PAR2 agonist, exhibited significant colon elongation and increased smooth muscle weight. In the 2‐F‐L‐a‐treated mice, PDGFRα, PAR2, and Ki‐67 coexpression was increased and PDGFRα and PAR2 mRNA and protein expression was significantly enhanced in the colonic smooth muscle layer. 2‐F‐L‐a also increased proliferation and PDGFRα expression in NIH/3T3 cells cultured in high glucose, while LY294002, a PI3K antagonist, decreased cell proliferation and PDGFRα expression. PI3K and Akt protein and mRNA expression and p‐Akt protein expression in diabetic and 2‐F‐L‐a‐treated mice were markedly reduced in colonic smooth muscle. 2‐F‐L‐a also reduced PI3K, Akt, and p‐Akt protein expression in NIH/3T3 cells, while the PI3K antagonist LY294002 increased this expression. The results indicate that PAR2 is involved in the proliferation of PDGFRα^+^ cells through the PI3K/Akt signaling pathway in the colon of STZ‐induced diabetic mice, which may contribute to the slow transit and constipation that are associated with diabetes.

## 1 INTRODUCTION

Diabetes is a universal metabolic sickness that seriously affects human health and can cause serious complications involving multiple organ systems; the incidence of diabetes worldwide has significantly increased in recent decades (Guariguata et al., 2014). Diabetes is a systemic disease that affects the nervous system, urinary system, cardiovascular system, and digestive system. Dysmotility of the gastrointestinal tract is a familiar chronic symptom accompanying diabetes mellitus (Feldman & Schiller, 1983; Ordog, 2008; Talley et al., 2001). The main manifestations of this disorder are low esophageal pressure, insufficient gastric motility, gastric emptying disorders, delayed gastrointestinal transit (gastroparesis), abdominal distension, nausea, vomiting, constipation, and diarrhea, and these symptoms seriously influence the patients’ quality of life, as well as the absorption of hypoglycemic drugs and blood glucose control. To date, the mechanisms underlying chronic constipation in diabetes mellitus patients remain unclear. Our previous study demonstrated that streptozotocin (STZ)‐induced type 1 diabetes mellitus results in slow transit constipation in a mouse model. The P2Y1 receptor and SK3 channel are located on platelet‐derived growth factor receptor α‐positive cells (PDGFRα^+^ cells). Our research further revealed that diabetes upregulated the expression of purinergic P2Y1 receptors and SK3 channels in colonic smooth muscle tissue and led to slow transit constipation in diabetic mice (Song et al., 2018). Therefore, it is necessary to explore the reason for the high abundance of PDGFRα^+^ cells in colon smooth muscle tissue caused by diabetes.

Recent studies have found that in the alimentary tract, in addition to interstitial cells of Cajal (ICCs), another type of interstitial cell is present, and these cells, referred to as PDGFRα^+^ cells, exhibit a morphology and properties similar to those of ICCs. In the gastrointestinal tract, ICCs and PDGFRα^+^ cells form gap junctions with smooth muscle cells to constitute a SIP (SMCs, ICCs, and PDGFRα^+^ cells) syncytium (Iino & Nojyo, 2009; Sanders, Koh et al., 2012; Sanders, Zhu et al., 2012). The SIP syncytium interacts with the enteric nervous system to regulate smooth muscle motility. ICCs and PDGFRα^+^ cells are distributed around the varicosities of nerve endings, and the neurotransmitters that are released by enteric nerves regulate smooth muscle motility through these two types of interstitial cells; for example, the excitatory and inhibitory effects of acetylcholine (Ach) and nitric oxide occur via ICCs, and the inhibitory effects of purinergic neurotransmitters occur via PDGFRα^+^ cells (Blair et al., 2014; Jiménez, 2015; Koh et al., 2012). Our previous study showed that in mice with STZ‐induced type I diabetes, colon lengthening significantly decreased colon motility and reduced colon transit and that colonic PDGFRα^+^ cells were significantly hyperplastic and hyperfunctional; however, the mechanism underlying diabetes‐induced PDGFRα^+^ cell proliferation was unclear (Lu et al., 2017; Song et al., 2018).

Members of the protease‐activated receptor (PAR) family, including PAR1, PAR2, PAR3, and PAR4, have seven transmembrane fragments and thus belong to the G protein‐coupled receptor family. PARs are widely distributed in epithelial cells, intestinal neurons, intestinal smooth muscle cells, ICCs, PDGFRα^+^ cells, and immune cells, such as lymphocytes, macrophages, and mast cells; these receptors are involved in regulation of digestive tract function under physiological and pathological conditions (Kawabata et al., 2008; Sato et al., 2006; Sung et al., 2015; Vergnolle, 2005; Zhao et al., 2014). For example, PARs participate in gastrointestinal motility, secretion of digestive mucosa, changes in epithelial permeability, regulation of visceral sensory function, and the pathogenesis of colitis. The colonic SIP syncytium mainly expresses PAR1 and PAR2, whereas PAR3 is expressed at significantly lower levels than PAR1 and PAR2, and the distribution of PAR1 and PAR2 in PDGFRα^+^ cells is much higher than that in ICCs and smooth muscle cells (Sung et al., 2015). Recent research has indicated that positive PAR2 expression promotes cell proliferation and migration and disrupts the quiescent condition of VSMCs in blood samples of atherosclerotic patients and in vascular smooth muscle cells of humans treated with oxidized LDL after transfection‐mediated PAR2 overexpression (Wei et al., 2019). Whether PARs are involved in the cell proliferation processes caused by various pathogenic factors, especially oxidative stress in diabetes, is unknown, and the answer to this question is of great significance for explaining the pathogenesis of many diabetic complications.

Based on these considerations, the present study aimed to investigate whether PAR2 participates in the proliferation of colonic PDGFRα^+^ cells in STZ‐induced diabetic mice.

## 2 MATERIALS AND METHODS

### 2.1 Ethics statement

This study was conducted according to the recommendations of the Guide for the Care and Use of Laboratory Animals of the Science and Technology Commission of the P.R.C. (STCC Publication No. 2, revised 1988). The protocol was approved by the Committee on the Ethics of Animals at Shanghai Jiao Tong University, and permit number 686‐2009 (Hu) was obtained.

### 2.2 STZ‐treated diabetic mouse model

Male ICR mice (5 weeks old) were maintained at 22°C under a 12‐h light/dark cycle and given free access to water and food. In total, 90 mice were included in this research. Mice were injected with STZ (Sigma Aldrich, 200 mg/kg), which was dissolved in ice‐cold citrate buffer (0.1 mol/L). Normal mice were injected with the same quantity of citrate buffer. Two months and 1 week later, we measured the blood glucose levels in STZ‐treated mice; if the blood glucose level of the mice exceeded 16.7 mmol/L, we confirmed the establishment of STZ‐induced diabetic mice.

### 2.3 PAR2 agonist treatment

Male ICR mice (3 weeks old) were maintained at 22°C under a 12‐h light/dark cycle and given free access to water and food. In total, 30 mice were included in this study. The mice in the 2‐F‐L‐a treatment group were intraperitoneally injected with 2‐F‐L‐a (1 μM/L; 0.2 ml/10 g; Tocris Bioscience) dissolved in double‐distilled water once a week, and the mice in the control group were injected with the same volume of saline once a week. Two weeks later, the mice were euthanized after 2‐F‐L‐a injection was completed.

### 2.4 NIH/3T3 fibroblast cell culture

NIH/3T3 fibroblasts were cultured in Dulbecco's modified Eagle's medium (DMEM; Gibco, Grand Island, NY, USA) containing 25 mmol/L glucose and supplemented with 100 mg/mL streptomycin, 10% heat‐inactivated bovine serum (BS), and 100 U/mL penicillin. The cells were incubated at 37°C with 5% CO_2_. The NIH/3T3 cells were seeded in plates with 50 mmol/L glucose (high glucose), and PI3K, PDGFRα, Akt, and p‐Akt expression was measured 12‐h and 24‐h later.

### 2.5 Preparation of colonic smooth muscle tissue

The diabetic mice were sacrificed by cervical dislocation, and the entire colon was removed and placed in Krebs solution with a carbonated mixture (95% O [Feldman & Schiller, 1983] and 5% CO_2_). The control mice were subjected to the same processing pattern. The Krebs solution contained the following components (all concentrations are in mmol/L): glucose, 11.5; NaCl, 121.9; KCl, 5.9; MgSO_4_, 1.2; NaHCO_3_, 15.5; CaCl_2_, 2.4; and KH_2_PO_4_, 1.2. The colon was cut along the mesentery, and colon length was measured before the colon pellets were flushed out using Krebs solution. The colon tissues were pinned to a rubber plate, and smooth muscle weight was measured after the submucosa and mucosa were carefully shifted under a microscope. The tissue samples were stored at −80°C.

### 2.6 Immunostaining

Tissue samples were embedded as frozen tissue blocks after dehydration in 20% sucrose, fixed with ice‐cold 4% paraformaldehyde for 6–8 h, and then cut into frozen sections with thicknesses of 8–10 µm at −20°C. The sections or NIH/3T3 cells were incubated in 0.1 M phosphate‐buffered saline (PBS) containing 10% normal goat serum for 2 h to block nonspecific binding and then cultured with a goat anti‐PDGFRα antibody (AF1062, 1:200; R&D Systems) and a rabbit anti‐Ki‐67 antibody (GB13030, 1:50; Wuhan Good Biotech Co.) or a rabbit anti‐PDGFRα antibody (1:1000; #3174; Cell Signaling Technology), and 24 h later a PAR2 (SAM11) antibody (1:50; sc‐13504; Santa Cruz Biotechnology, Inc.) in Triton X‐100 (Sigma Aldrich, 0.5%; USA) at 4°C. The samples were washed with 0.1 M PBS for 30 min and then incubated at room temperature with Cy3‐conjugated anti‐goat IgG (1:300; GB21404, Wuhan Good Biotech Co.), Alexa Fluor 488‐conjugated goat anti‐rabbit IgG (Jackson ImmunoResearch, 1:100), Alexa Fluor 488‐conjugated goat anti‐mouse IgG (A0428, 1:200; Beyotime Institute of Biotechnology) and DAPI (2 h). Images were obtained using a fluorescence microscope (ZEISS Axiovert 200).

### 2.7 RNA isolation and reverse‐transcription quantitative PCR

According to the manufacturer's directions, we extracted total RNA from the smooth muscle layers of the colon using an RNA simple Total RNA kit (Tian gen) and then synthesized first‐strand cDNA using a PrimeScript RT Reagent Kit (Takara) with gDNA Eraser. Reverse‐transcription quantitative PCR using FastStart Universal SYBR Green MasterMix (Roche) was performed on a 7500 Real‐Time PCR System (Applied Biosystems) with ad hoc primers. The ∆CT method was utilized to determine the expression of the target genes relative to that of the endogenous control. The primer sequences that we used are as follows:

PDGFRα: F‐CTGGTGGTCATTTGGAAGC and R‐GGAGTCGTAAGGCAACTG;

PAR2: F‐CACCTGGCAAGAAGGCTAA and R‐ CAACTGGACTGAAGCTCTAC;

PI3K: F‐AGATGAGACAGCCAGACT and R‐TCTTCAAGCCTGAGGTTTCCTA;

GAPDH: F‐TGCGACTTCAACAGCAACTC and R‐ATGTAGGCAATGAGGTCCAC.

### 2.8 Western blot analysis

Protein samples were obtained from the colon muscle layers lysed in radioimmunoprecipitation assay (RIPA) buffer (P0013, 1:10; Beyotime Chemical Co.) and 1:100 PMSF solution. The suspensible material was centrifuged for 15 min at 14010 g and 4°C, combined with 4× loading buffer, and stewed for 5 min in a boiling water bath. Equal amounts of protein (30 μg/lane) were separated on 10% or 7.5% SDS‐PAGE gels and subsequently transferred to polyvinylidene difluoride (PVDF) membranes. We incubated the PVDF membranes, which were blocked with 5% nonfat milk in 0.1% Tris‐buffered saline/Tween 20 (TBST), overnight with primary antibodies at 4°C and then with secondary antibodies for 2 h at room temperature. The antibody dilutions and antibodies used were as follows: PAR2 (SAM11) antibody (1:500; sc‐13504; Santa Cruz Biotechnology, Inc.), PDGFRα rabbit antibody, PI3 kinase p110α (C73F8) rabbit antibody, protein kinase B (Akt) rabbit antibody, phospho‐Akt (Ser473) rabbit antibody, glyceraldehyde 3‐phosphate dehydrogenase (GAPDH) rabbit antibody, anti‐rabbit IgG, HRP‐linked antibody, and anti‐mouse IgG, HRP‐linked antibody (all 1:1000; #3174; #4249; #4691; #4060; #2118; #7074; #7076; Cell Signaling Technology), and anti‐tubulin antibody (1:1000; AT819; Beyotime Chemical Co).

### 2.9 Cell survival assay

Living cells were counted using Cell Counting Kit‐8 (Beyotime), which combines monosodium salt, 1‐methoxy PMS [5‐methylphenazinium, methylsulfate], and WST‐8 [2‐ (2–methoxy‐4–nitrophenyl)‐3‐(4–nitrophenyl)–5‐(2,4 disulfophenyl)‐2*H*‐tetrazolium]. Briefly, the cells were seeded in 96‐well tissue culture plates for 24 h and incubated with control medium or drug. Next, 20 μl of kit reagent was added and incubated with the cells for 1 h in 5% CO_2_ at 37°C. Then, the formazan dye was quantified by detecting the optical density at 480 nm using an ELISA plate reader. The absorbance was correlated with the number of metabolically active cells.

### 2.10 Statistical analysis

The results were statistically analyzed using the computer program GraphPad Prism6 or ImageJ software. We chose the image tab and then clicked the option merge channels under the color tab on ImageJ software to merge pictures. The data are expressed as the means ± standard error of the mean (SEM). Among the multiple groups, quantitative data were examined using one‐way ANOVA with Bonferroni's post hoc test. Unpaired Student's *t*‐tests were used to determine the significance of differences between two groups. The data were analyzed at a *p*‐value <0.05 level of significance.

## 3 RESULTS

### 3.1 PAR2 and PDGFRα coexpression in colonic muscle tissue of diabetic mice

**FIGURE 1:**
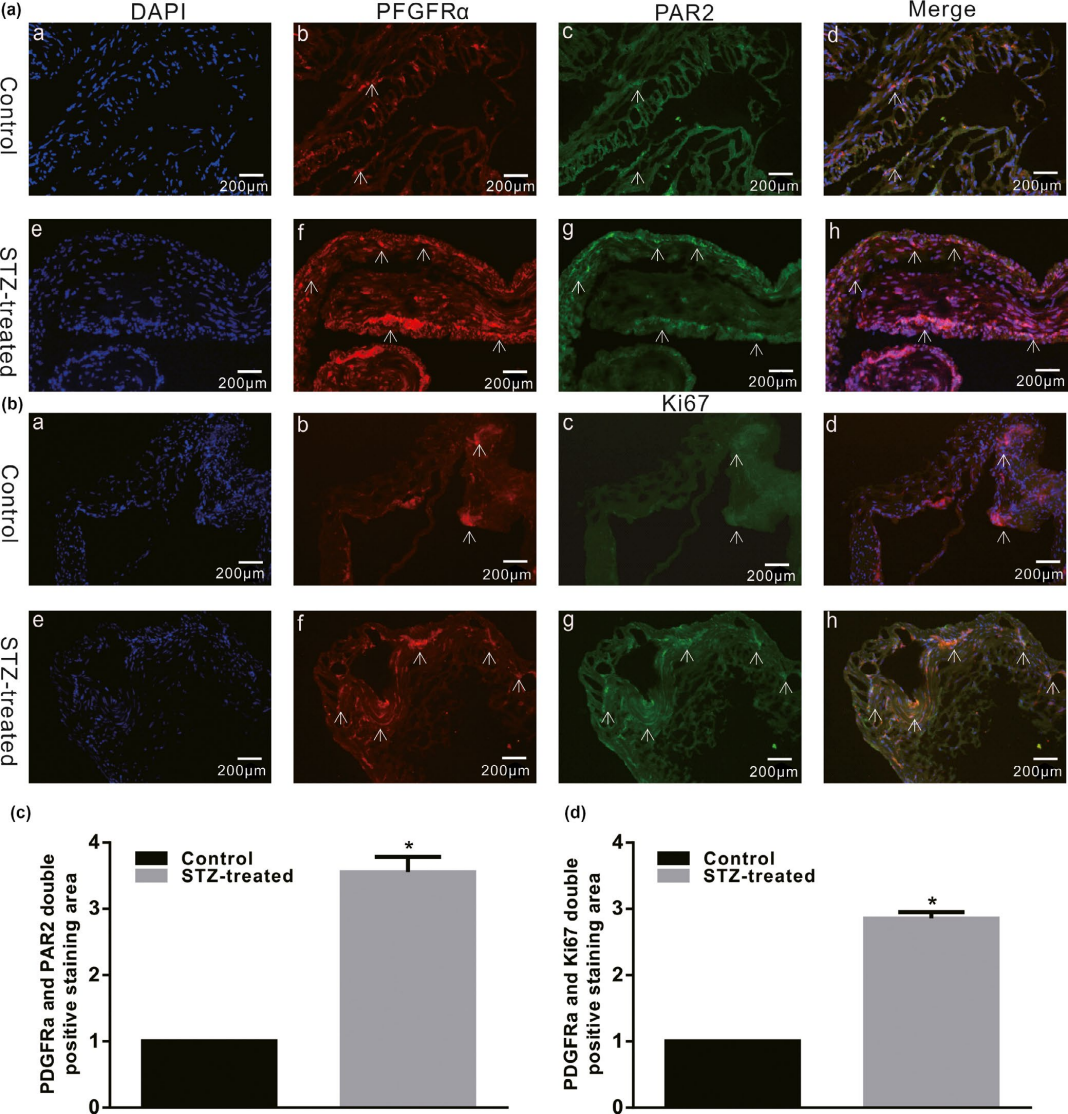
PAR2, PDGFRα, and Ki‐67 expression in the colon muscle tissues of STZ‐induced diabetic mice. Immunofluorescence staining showed substantially different Ki‐67 and PDGFRα coexpression levels (A) and PDGFRα and PAR2 coexpression levels (B) in the colon smooth muscle layers between the control and STZ‐induced diabetic mice (bar = 100 μm). (C and D) The double‐positive staining region was detected using ImageJ software (*n* = 4; *p < 0.05)

PDGFRα^+^ cells play a vital function in regulation of colonic motility by handling potassium channels, such as SK3 channels. SK3 channels are located on PDGFRα^+^ cells. In addition, the PAR2 ligand binds to PAR2 to activate SK3 in PDGFRα^+^ cells and hyperpolarize the cell membrane potential, which induces SIP‐mediated smooth muscle relaxation. However, whether PAR2 contributes to colonic motility disorder induced by diabetes is currently unclear, and thus, coexpression of PDGFRα and PAR2 in colonic muscle tissues was investigated in STZ‐induced diabetic mice. The data indicated that PDGFRα and PAR2 coexpression was significantly increased in the colonic smooth muscle layers of STZ‐treated mice (Figure 1A,C, *p* < 0.05, *n* = 4). Moreover, to further clarify the proliferation state of PDGFRα^+^ cells in colon muscle tissue in diabetes, Ki‐67 expression in colon muscle tissue was measured in STZ‐treated mice. The results indicated that PDGFRα and Ki‐67 coexpression was significantly increased in STZ‐treated mice (Figure 1B,D, *p* < 0.05, *n* = 4).

**FIGURE 2:**
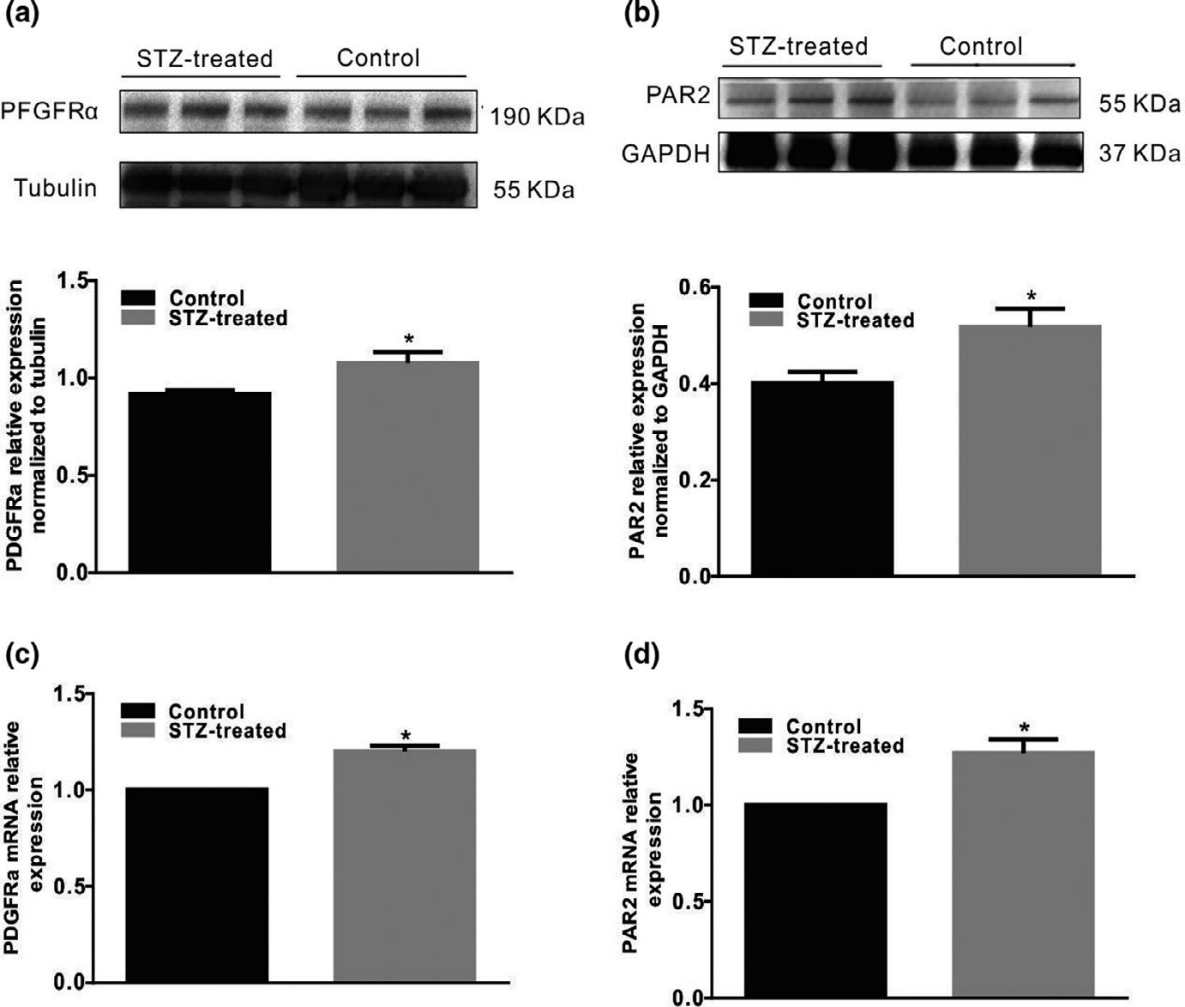
PDGFRα and PAR2 expression levels in the colonic muscle tissues of diabetic mice. Western blot analysis of PDGFRα (a) and PAR2 (b) expression in normal and STZ‐treated diabetic mice. Ratio of PDGFRα and PAR2 to tubulin or GAPDH compared with that in the control mice. (*n* = 5, *p < 0.05). Quantitative RT‐PCR was used to analyze PDGFRα (c) and PAR2 (d) expression in the colon muscle layers of normal and STZ‐induced diabetic mice. The results were normalized to GAPDH expression and the normal mice (% of GAPDH and normalized to the data of the normal mice; PAR2, *p < 0.05, *n* = 5; PDGFRα, *p < 0.05, *n* = 5)

We observed the abundance of PDGFRα^+^ cells with reference to the experimental methods of Ino et al. (2009) and explored the difference in PDGFRα expression in the colon smooth muscle layer of control and diabetic mice. In STZ‐treated diabetic mice, PDGFRα protein expression was found to be significantly increased to 117 ± 5% (Figure 2a, *p* < 0.05, *n* = 5) in the colonic smooth layer. RT‐PCR results showed that PDGFRα gene expression was increased to 120 ± 3% (Figure 2c, *p* < 0.05, *n* = 5) in STZ‐treated diabetic mice. PAR2 protein and gene expression were also measured, and the results showed that the PAR2 protein and F2rl1 gene (which encodes the PAR2 protein) expression levels were significantly increased to 135 ± 8% (Figure 2b, *p* < 0.05, *n* = 5) and 127 ± 7% (Figure 2d, *p* < 0.05, *n* = 5), respectively, in the STZ‐induced diabetic mice. These phenomena demonstrate that diabetes significantly increased PDGFRα and PAR2 coexpression in the colonic muscle layer.

### 3.2 Effects of PAR2 agonist on colon length and smooth muscle weight

**FIGURE 3:**
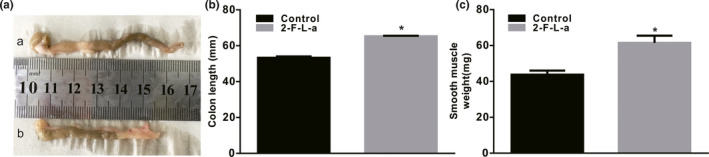
Change in colonic length in 2‐F‐L‐a‐treated mice. (A) Comparison of the colon length between the control and 2‐F‐L‐a‐treated mice. (B) Summarized data showing the colon length in control and 2‐F‐L‐a‐treated mice (*p < 0.05; *n* = 15). (C) Summarized data showing the smooth muscle weight in the control and STZ‐treated mice. The data are shown as the mean ± SEM. (*p < 0.05; *n* = 15)

PAR2 signaling is involved in cell proliferation and migration, such as promoting vascular endothelial cell proliferation/migration and increasing proangiogenic factors. Whether PAR2 signaling contributes to PDGFRα^+^ cell proliferation in diabetic mice is unclear. After two 2 weeks, the mice were pretreated with 2‐F‐L‐a, a PAR2 agonist, as described in a previous report (Arderiu et al., 2016; McGuire et al., 2004; Zhu et al., 2006), and changes in colon length and smooth muscle weight were measured. In the 2‐F‐L‐a‐treated group, the colons were significantly elongated, and colonic lengths were increased to 65.27 ± 0.30 mm from 53.33 ± 0.67 mm in the control group (Figure 3Aa,B, *p* < 0.05, *n* = 15). The weight of the colonic smooth muscle from 2‐F‐L‐a‐treated mice was significantly increased, with an average weight of 61.47 ± 1.04 mg compared with an average weight of 43.93 ± 0.59 mg (*n* = 15; *p* < 0.05; Figure 3C) in the control mice. These results suggest that PAR2 may be involved in diabetes‐induced colon PDGFRα^+^ cell proliferation.

### 3.3 PAR2 agonist increased colonic PDGFRα expression in mice

**FIGURE 4:**
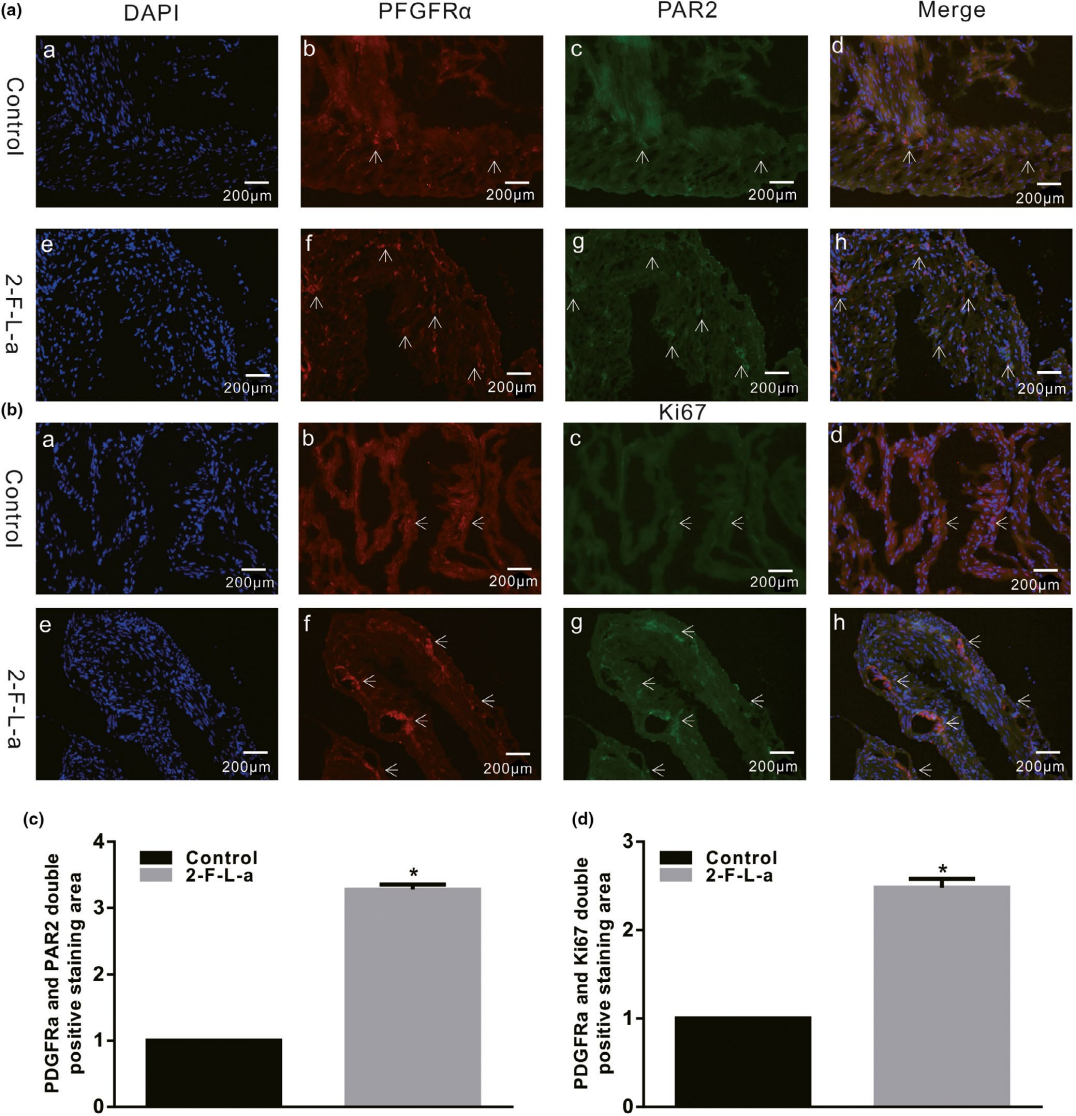
PDGFRα, Ki‐67, and PAR2 coexpression in the 2‐F‐L‐a‐treated colonic muscle tissues. (A and B) Immunofluorescence staining revealed different PDGFRα and Ki‐67 coexpression levels and PDGFRα and PAR2 coexpression levels in the colon smooth muscle layers between the control and 2‐F‐L‐a‐treated mice (bar = 100 μm). The double‐positive staining region was detected using ImageJ software. (2‐F‐L‐a: PAR2 agonist; *n* = 4; *p < 0.05)

To determine the relationship between PAR2 and diabetes‐induced PDGFRα^+^ cell proliferation, PDGFRα and PAR2 coexpression was observed in the colonic muscle tissues of 2‐F‐L‐a‐treated mice. The PDGFRα and PAR2 coexpression level was assessed by measuring the double‐positive staining area using ImageJ software, and the results showed that PDGFRα and PAR2 coexpression was much higher in the 2‐F‐L‐a‐treated group than in the control group (Figure 4A,C, *n* = 4, *p* < 0.05). Furthermore, at different cell cycle stages, Ki‐67 is a recognized cell proliferation indicator; thus, Ki‐67 expression was observed in colon muscle tissue to assess the PDGFRα^+^ cell proliferation status in 2‐F‐L‐a‐treated mice. The expression of colonic PDGFRα and Ki‐67 in 2‐F‐L‐a‐treated mice was significantly increased, and evaluation of the double‐positive staining area showed that PDGFRα and Ki‐67 coexpression was evidently increased (Figure 4B,D, *n* = 4, *p* < 0.05).

**FIGURE 5:**
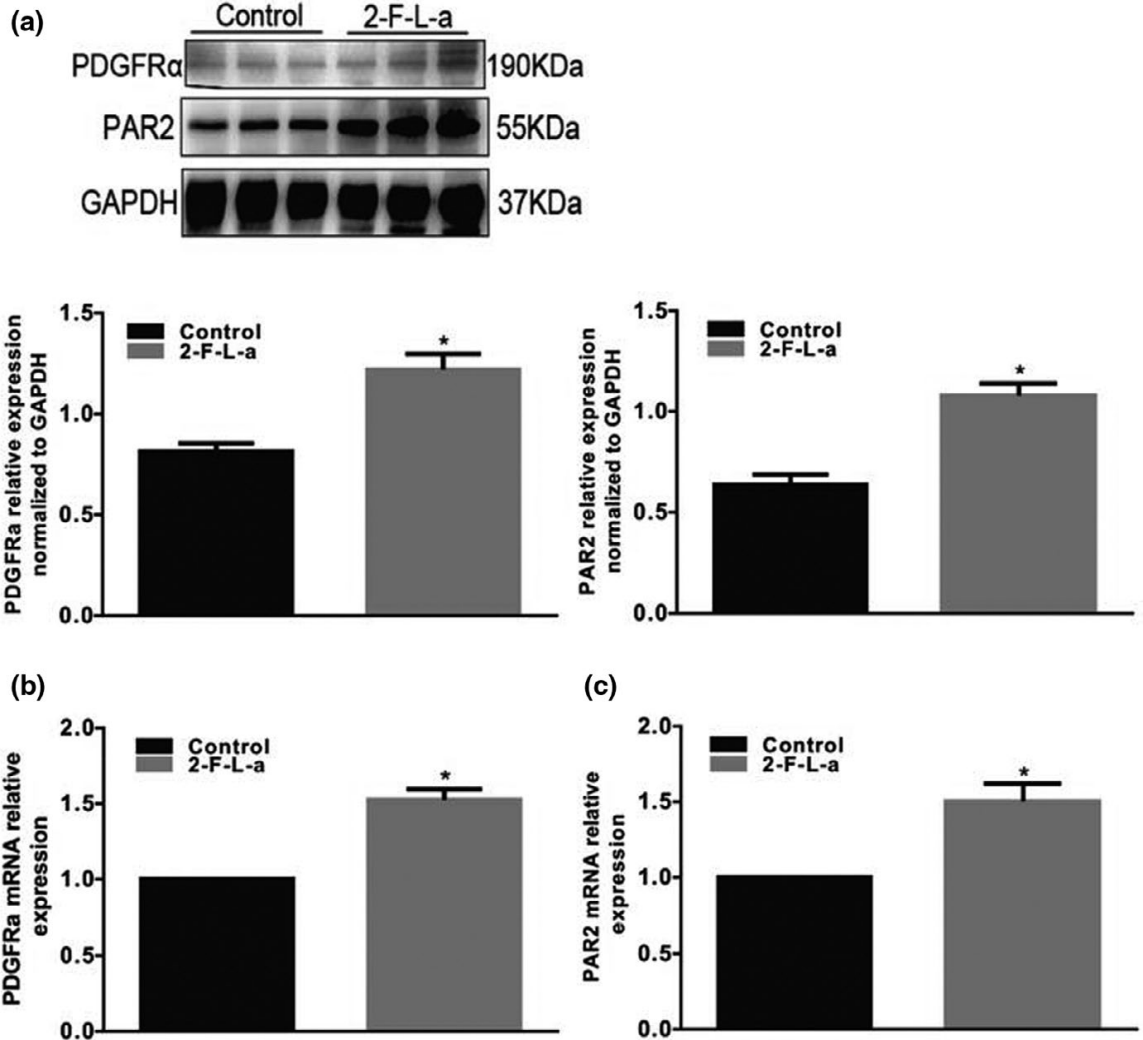
PDGFRα and PAR2 coexpression levels in the colon muscle tissues of 2‐F‐L‐a‐treated mice. The expression of PDGFRα (a) and PAR2 (b) in normal and 2‐F‐L‐a‐induced mice was analyzed using western blotting. GAPDH served as a loading control, and densitometric values are shown. (2‐F‐L‐a: PAR2 agonist; *n* = 5; *p < 0.05). Quantitative RT‐PCR was used to analyze PDGFRα (c) and PAR2 (d) expression in the colon muscle layers of normal and 2‐F‐L‐a‐induced mice. The results were normalized to GAPDH expression and the expression level in normal mice (% of GAPDH and normalized to the data of the normal mice; 2‐F‐L‐a: PAR2 agonist; *n* = 4; *p < 0.05)

In addition, the PDGFRα and PAR2 protein and mRNA expression levels in the colonic muscle tissue of 2‐F‐L‐a‐treated mice were evaluated. Compared with the control mice, the 2‐F‐L‐a‐treated mice showed evidently upregulated PDGFRα and PAR2 protein and mRNA expression (Figure 5a–d, *p* < 0.05, *n* = 4). These results suggest that PAR2 may be involved in the proliferation of colonic PDGFRα^+^ cells in mice.

### 3.4 The effect of PAR2 agonist on PI3K/Akt expression in the colon of diabetic mice

**FIGURE 6:**
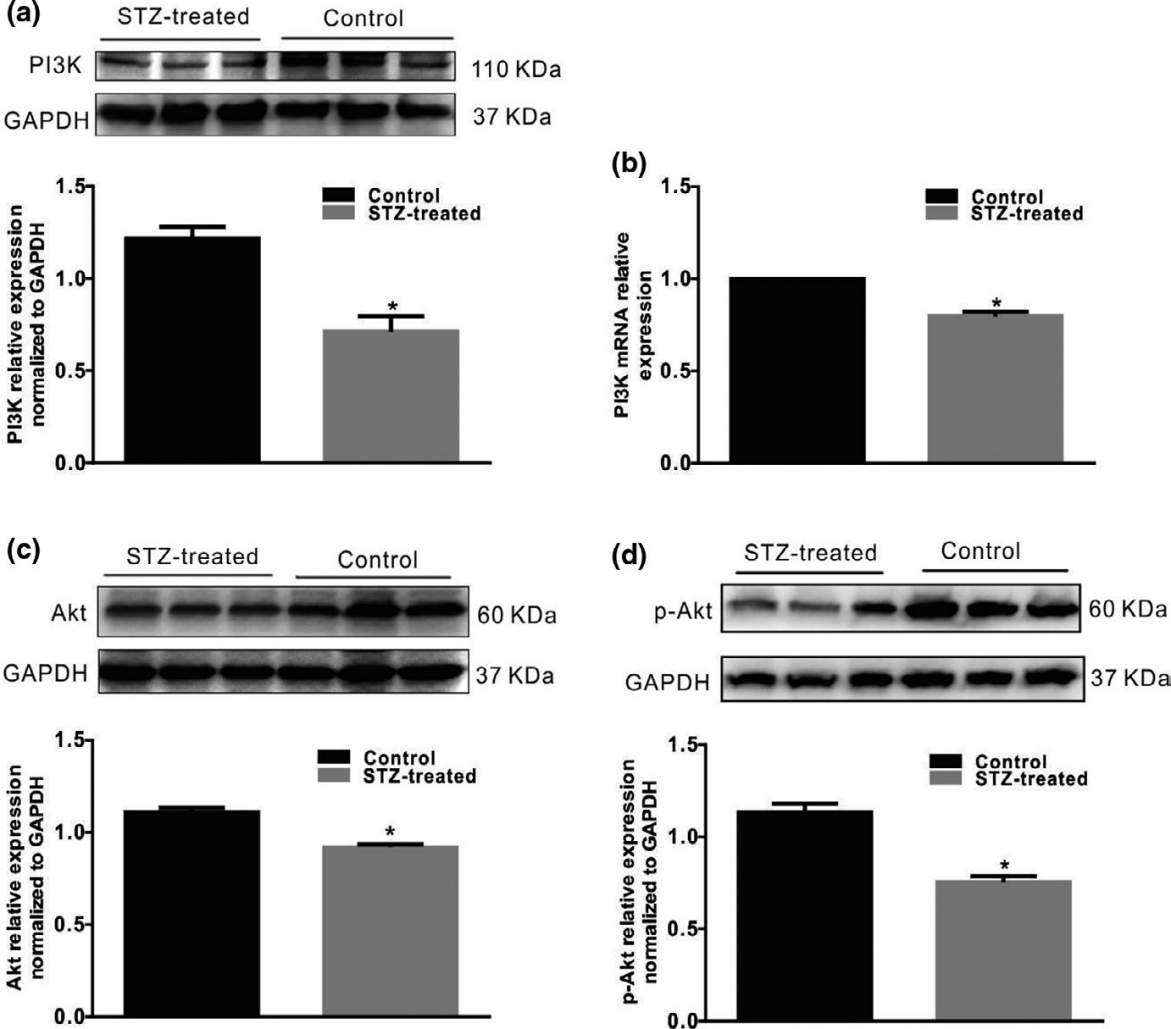
PI3K/Akt expression in colonic muscle tissues from diabetic mice. (a, c, d) PI3K/Akt expression in normal and STZ‐induced diabetic mice were analyzed using western blotting. GAPDH served as a loading control, and densitometric values are shown. (*n* = 5, *p < 0.05). (b) PI3K expression in the colon muscle layers of normal and STZ‐induced diabetic mice were analyzed via quantitative RT‐PCR. The results were normalized to GAPDH expression and the expression level in normal mice. (% of GAPDH and normalized to data of the normal mice; *p < 0.05; *n* = 5)

**FIGURE 7:**
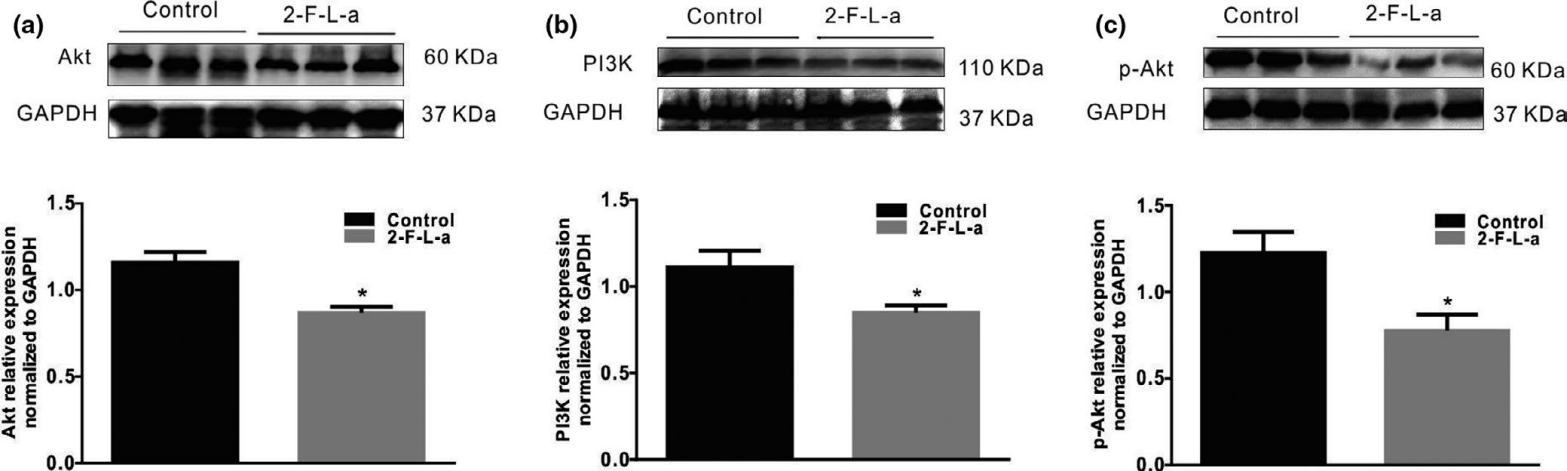
PI3K/Akt expression in colonic muscle tissues of 2‐F‐L‐a‐induced mice. (a, b and c) Western blot analysis of PI3K, Akt, and p‐Akt expression in the control and 2‐F‐L‐a‐treated mice. The data were analyzed using densitometric quantification (% of GAPDH and normalized to the data of the control mice; *n* = 5, *p < 0.05)

As the main downstream effector of growth factors, the PI3K/Akt pathway is involved in many intracellular biological functions. To investigate whether the PI3K/Akt signaling pathway mediates the process by which PAR2 induces PDGFRα cell proliferation in the colon of STZ‐treated diabetic mice, the effect of a PAR2 agonist on the colonic PI3K/Akt signaling pathway was investigated in diabetic mice. PI3K protein and mRNA expression levels were significantly decreased in STZ‐induced diabetic mice (Figure 6a,b, *n* = 6, *p* < 0.05). The total and phosphorylated Akt protein levels were significantly downregulated in the colonic muscle tissues of the diabetic group (Figure 6c,d, *p* < 0.05, *n* = 6). Similarly, in the 2‐F‐L‐a‐treated group, PI3K protein expression was also importantly reduced (Figure 7b, *n* = 6, *p* < 0.05), and the total and phosphorylated Akt protein levels were evidently downregulated in the colonic muscle tissues (Figure 7a,c, *p* < 0.05, *n* = 6). These data suggest that PAR2 is involved in diabetes‐induced colon PDGFRα^+^ cell proliferation processes, which may be mediated by PI3K/Akt signaling pathways.

### 3.5 The effect of a PAR2 agonist on PDGFRα^+^ NIH/3T3 cell proliferation

**FIGURE 8:**
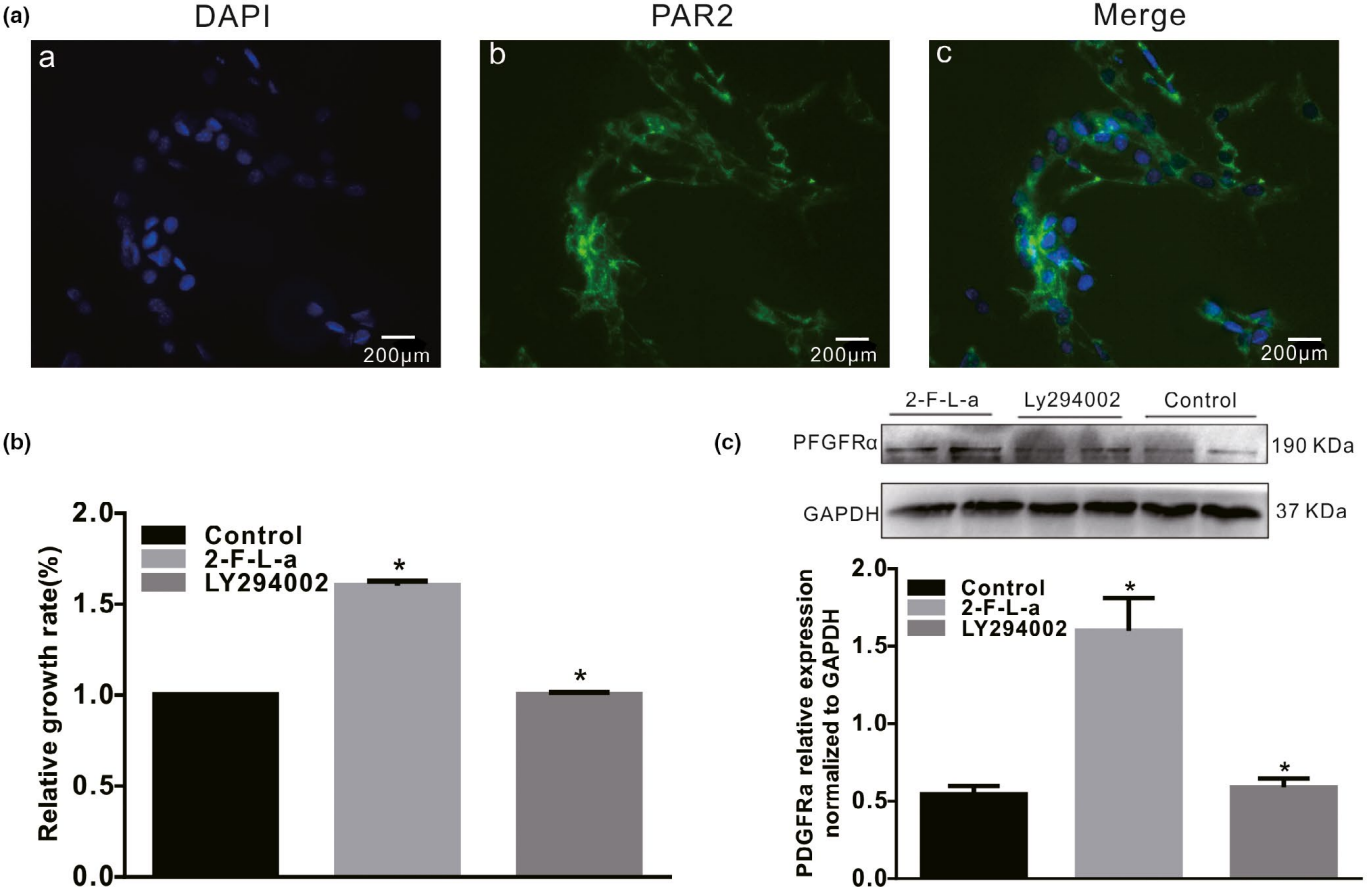
PAR2 expression in NIH/3T3 cells. (A) Immunofluorescence staining revealed the PAR2 expression levels (bar = 100 μm) in NIH/3T3 cells. (B) LY294002 inhibited the 2‐F‐L‐a‐induced proliferation of NIH/3T3 cells. The graph summarizes the data showing the relative growth rates of the control, 2‐F‐L‐a‐treated and 2‐F‐L‐a plus LY294002‐treated NIH/3T3 cells. (C) Western blot analysis of PDGFRα protein expression in the control, 2‐F‐L‐a‐treated, and 2‐F‐L‐a plus LY294002‐treated NIH/3T3 cells. The graph summarizes the data showing PDGFRα protein expression. (2‐F‐L‐a: PAR2 agonist; LY294002: PI3K antagonist; *n* = 6; *p < 0.05)

PDGFRα^+^ cells are called “fibroblast‐like cells” (FLCs) by morphologists, and NIH/3T3 cells are established from mouse embryonic fibroblasts; thus, NIH/3T3 cells also express PDGFRα. Moreover, the PAR2 (Figure 8A, *n* = 6) and PDGFRα proteins (Figure 8C) are expressed in NIH/3T3 cells. Therefore, NIH/3T3 cells are suitable for use as an in vitro model to examine the relationship between PAR2 and PDGFRα^+^ cell proliferation. NIH/3T3 cells cultured in high glucose were incubated with the PAR2 agonist 2‐F‐L‐a at 1 μmol/L for 24 h, and the NIH/3T3 cells exhibited significant growth; however, when the NIH/3T3 cells were incubated with 1 μmol/L 2‐F‐L‐a and LY294002, a PI3K antagonist, for 24 h, the 2‐F‐L‐a‐induced cell growth was significantly inhibited (Figure 8B, *n* = 6, *p* < 0.05). Similarly, after treatment with 1 μmol/L 2‐F‐L‐a, PDGFRα protein expression was significantly increased, while after treatment with 1 μmol/L 2‐F‐L‐a plus LY294002 for 24 h, PDGFRα protein expression was significantly reduced in whole cell extracts (Figure 8C,D, *n* = 6, *p* < 0.05). These results suggest that PAR2 promotes PDGFRα^+^ NIH/3T3 cell proliferation.

**FIGURE 9:**
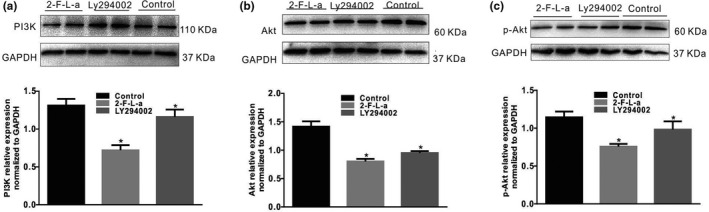
PAR2 expression in NIH/3T3 cells. Immunofluorescence staining revealed the PAR2 expression levels in NIH/3T3 cells (*n* = 6). LY294002 inhibited the 2‐F‐L‐a‐induced proliferation of NIH/3T3 cells. The graph summarizes the data showing the relative growth rates of the control, 2‐F‐L‐a‐treated, and 2‐F‐L‐a plus LY294002‐treated NIH/3T3 cells. (2‐F‐L‐a: PAR2 agonist; LY294002: PI3K antagonist; *n* = 6; *p < 0.05)

Next, the PI3K, Akt, and p‐Akt protein expression levels in NIH/3T3 cells were found to be significantly decreased by treatment with 1 μmol/L 2‐F‐L‐a, while treatment with 2‐F‐L‐a and LY294002 for 24 h significantly increased the PI3K, Akt, and p‐Akt protein expression levels in NIH/3T3 cells (Figure 9, *n* = 4, *p* < 0.05). These results suggest that PAR2 participates in NIH/3T3 cell proliferation, which is mediated by PI3K/Akt signaling.

## 4 DISCUSSION

In recent reports, colonic dysmotility has been shown to occur in diabetes patients, and many patients exhibit constipation (Camilleri et al., 2017; Lembo & Camilleri, 2003). The patterns of colonic motility and transit are regulated by the enteric nervous system and interstitial cells, such as ICCs and PDGFRα^+^ cells. The purinergic nerve is an important inhibitory nerve in colon smooth muscle, while purinergic neurotransmitters activate SK3 channels through P2Y1 receptors on PDGFRα^+^ cells and mediate the inhibitory effect of purinergic nerves on colon smooth muscle (Baker et al., 2013). PAR2 expression in PDGFRα^+^ cells is much higher in the colon than in ICCs and smooth muscle cells; however, in normal smooth muscle, PAR2 is activated by trypsin, which can lead to APAMIN‐sensitive outward potassium current activation (Sung et al., 2015). Our previous study revealed that the number of PDGFRα^+^ cells was significantly increased and that purine neurotransmitter/P2Y1/SK3 was upregulated in the diabetic colon, which resulted in colonic slow transit (Camilleri et al., 2017). However, at present, there are no reports that diabetes can affect colon PDGFRα^+^ cell proliferation induced by PAR2. The purpose of this study was to determine whether PAR2 is involved in the proliferation of PDGFRα^+^ cells under diabetic conditions.

Regulatory disorders of PAR‐2 activation can lead to a large number of common human diseases, especially due to the ability of PAR‐2 receptors to induce key inflammatory signaling pathways (Coughlin & Camerer, 2003). Moreover, Ki‐67 is a nuclear antigen that is, generally used as an indicator of cell proliferation because Ki‐67 is expressed only during the G1, S, and G2 mitotic phases of the cell cycle but not in the G0 resting phase (Yuan et al., 2015). In this study, we first explored the interaction between PAR2 and colon PDGFRα expression in diabetic mice and preliminarily judged whether PAR2 affects PDGFRα^+^ cells. The results showed that PDGFRα and Ki‐67 coexpression was significantly increased in the diabetes and PAR2 agonist groups compared with the control group (Figures 1 and 4). Furthermore, the PDGFRα and PAR2 mRNA expression levels were significantly upregulated in the diabetic and PAR2 agonist‐treated mice (Figures 2 and 5), and the PDGFRα and PAR2 proteins showed similar expression. The above results strongly suggest that increased coexpression of PDGFRα and PAR2 in the colon of diabetic or PAR2 agonist‐treated mice may be associated with the number of PDGFRα^+^ cells. Previous evidence indicates that binding of a PAR2 agonist to PAR2 stimulates cell proliferation‐mediated PI3K/Akt signaling (Du et al., 2017; Kawabata, 2003). In the present study, the expression levels of PI3K, Akt, and p‐Akt in colonic muscle tissues were significantly decreased in both STZ‐ and PAR2‐treated mice (Figures 6 and 7). The above data suggest that the proliferation of colonic PDGFRα^+^ cells is significantly increased in the STZ‐induced mouse diabetic model and is accompanied by upregulation of PAR2 expression. PAR2 is involved in PDGFRα^+^ cell proliferation through its effect on the PI3K/Akt signaling pathway, leading to an imbalance in the function of ICCs and PDGFRα^+^ cells in the SIP syncytium, which induces diabetic slow transit constipation.

We propose that PAR2 signaling is linked to cell homeostasis and mitogenic signaling pathways in the NIH/3T3 fibroblast cell line. After NIH/3T3 cells were cultured in high glucose and incubated with a PAR2 agonist for 24 h, the cells exhibited significant growth; however, incubation of the NIH/3T3 cells with the PAR2 agonist and LY294002, a PI3K antagonist, significantly inhibited the growth‐promoting effect (Figure 8). Similarly, PDGFRα protein expression in NIH/3T3 cells was significantly increased by PAR2 agonist treatment, while PDGFRα protein expression was significantly decreased by PAR2 agonist and LY294002 treatment (Figure 8). These data suggest that PAR2 may be involved in NIH/3T3 cell proliferation and PDGFRα expression, as PAR2 is associated with increased protease‐mediated hydrolysis, both in vitro and in vivo. In response to protease hydrolysis, PAR2 proteins are activated, thus contributing to a decrease in PI3K/Akt activity. The decrease in PI3K/Akt activity is associated with FOXO dephosphorylation, and dephosphorylated FOXO translocates into the nucleus and acts as a transcription factor (Accili & Arden, 2004; Dobson et al., 2011; Kawabata et al., 2005; Lee & Dong, 2017). Next, it was observed that the PI3K, Akt, and p‐Akt protein expression levels in NIH/3T3 cells treated with the PAR2 agonist were markedly decreased, while the PI3K, Akt, and p‐Akt protein expression levels in NIH/3T3 cells treated with the PAR2 agonist and LY294002 were markedly increased (Figure 9). These data suggest that PAR2 is involved in the process of NIH/3T3 cell proliferation, which is mediated by PI3K/Akt signaling.

In summary, diabetes induces the proliferation of PDGFRα^+^ cells in the colon, leading to an imbalance between ICCs and PDGFRα^+^ cells in the colonic SIP syncytium, which leads to diabetic slow transit constipation. The hyperfunction of colonic PAR2 induced by diabetes is involved in the process of colonic PDGFRα^+^ cell proliferation, which is mediated by the PI3K/Akt signaling pathway. It is also possible that PAR2 upregulation may be due to the inflammatory stress caused by hyperglycemia. Colonic transit is regulated by the enteric nerve, and this process is mediated by interstitial cells, such as ICCs and PDGFRα^+^ cells. The diabetes‐induced increase in PAR2 can promote colonic PDGFRα^+^ cell proliferation and can stimulate SK3 channel activation on the cell membrane of PDGFRα^+^ cells, which may lead to colonic motility disorders. Therefore, PAR2 and PDGFRα^+^ cells may be new targets for clinical treatment of diabetic colonic transit disorders.

## CONFLICT OF INTERESTS

The authors have no competing interests.

## AUTHOR CONTRIBUTIONS

Yu‐Jia and Jun‐Ping: conception and design of the experiments; collection, analysis and interpretation of the data; drafting of the article; and critical revision of the paper for important intellectual content. Xu and Hong‐Li: collection, analysis, and interpretation of the data. Ni‐Na and Han‐Yue: conception and design of the experiments. Wen‐Xie: conception, design of the experiments, and analysis and interpretation of the data. Jie: conception and design of the experiments; analysis and interpretation of the data; and critical revision of the paper for important intellectual content. All the authors approved the final version of the manuscript.

## Data Availability

The datasets used and/or analyzed in the current study are available from the corresponding author upon reasonable request.

## References

[phy215099-bib-0001] Accili, D. , & Arden, K. C. (2004). FoxOs at the crossroads of cellular metabolism, differentiation, and transformation. Cell, 117(4), 421–426. 10.1016/S0092-8674(04)00452-0 15137936

[phy215099-bib-0002] Arderiu, G. , Espinosa, S. , Pena, E. , Aledo, R. , & Badimon, L. (2016). PAR2‐SMAD3 in microvascular endothelial cells is indispensable for vascular stability via tissue factor signaling. Journal of Molecular Cell Biology, 8(3), 255–270. 10.1093/jmcb/mjv065 26658897

[phy215099-bib-0003] Baker, S. A. , Hennig, G. W. , Salter, A. K. , Kurahashi, M. , Ward, S. M. , & Sanders, K. M. (2013). Distribution and Ca(2+) signalling of fibroblast‐like (PDGFR(+)) cells in the murine gastric fundus. Journal of Physiology, 591, 6193–6208. 10.1113/jphysiol.2013.264747 PMC3892471 24144881

[phy215099-bib-0004] Blair, P. J. , Rhee, P.‐L. , Sanders, K. M. , & Ward, S. M. (2014). The significance of interstitial cells in neurogastroenterology. Journal of Neurogastroenterology and Motility, 20, 294–317. 10.5056/jnm14060 24948131 PMC4102150

[phy215099-bib-0005] Camilleri, M. , Ford, A. C. , Mawe, G. M. , Dinning, P. G. , Rao, S. S. , Chey, W. D. , Simrén, M. , Lembo, A. , Young‐Fadok, T. M. , & Chang, L. (2017). Chronic constipation. Nature Reviews Disease Primers, 3, 17095. 10.1038/nrdp.2017.95 29239347

[phy215099-bib-0006] Coughlin, S. R. , & Camerer, E. (2003). PARticipation in inflammation. Journal of Clinical Investigation, 111, 25–27. 10.1172/JCI17564 PMC151847 12511583

[phy215099-bib-0007] Dobson, M. , Ramakrishnan, G. , Ma, S. , Kaplun, L. , Balan, V. , Fridman, R. , & Tzivion, G. (2011). Bimodal regulation of FoxO3 by AKT and 14‐3‐3. Biochimica Et Biophysica Acta (BBA) ‐ Molecular Cell Research, 1813(8), 1453–1464. 10.1016/j.bbamcr.2011.05.001 21621563 PMC3237389

[phy215099-bib-0008] Du, C. , Zhang, T. , Xiao, X. , Shi, Y. , Duan, H. , & Ren, Y. (2017). Protease‐activated receptor‐2 promotes kidney tubular epithelial inflammation by inhibiting autophagy via the PI3K/Akt/mTOR signalling pathway. The Biochemical Journal, 474(16), 2733–2747. 10.1042/BCJ20170272 28694352

[phy215099-bib-0009] Feldman, M. , & Schiller, L. R. (1983). Disorders of gastrointestinal motility associated with diabetes mellitus. Annals of Internal Medicine, 98, 378–184. 10.7326/0003-4819-98-3-378 6402969

[phy215099-bib-0010] Guariguata, L. , Whiting, D. R. , Hambleton, I. , Beagley, J. , Linnenkamp, U. , & Shaw, J. E. (2014). Global estimates of diabetes prevalence for 2013 and projections for 2035. Diabetes Research and Clinical Practice, 103, 137–149. 10.1016/j.diabres.2013.11.002 24630390

[phy215099-bib-0011] Iino, S. , & Nojyo, Y. (2009). Immunohistochemical demonstration of c‐Kit‐negative fibroblast‐like cells in murine gastrointestinal musculature. Archives of Histology and Cytology, 72, 107–115. 10.1679/aohc.72.107 20009347

[phy215099-bib-0012] Ino, S. , Horiguchi, K. , Horiguchi, S. , & Nojyo, Y. (2009). c‐Kit‐negative fibroblast‐like cells express platelet‐derived growth factor receptor alpha in the murine gastrointestinal musculature. Histochemistry and Cell Biology, 131(6), 691–702. 19280210 10.1007/s00418-009-0580-6

[phy215099-bib-0013] Jiménez, M. (2015). Platelet‐derived growth factor receptor‐*α*‐positive cells: New players in nerve‐mediated purinergic responses in the colon. Journal of Physiology, 593, 1765–1766. 10.1113/JP270259 PMC4405736 25871557

[phy215099-bib-0014] Kawabata, A. (2003). Physiological functions of protease‐activated receptor‐2. Nihon Yakurigaku Zasshi, 121(6), 411–420. 10.1254/fpj.121.411 12835535

[phy215099-bib-0015] Kawabata, A. , Matsunami, M. , & Sekiguchi, F. (2008). Gastrointestinal roles for proteinase‐activated receptors in health and disease. British Journal of Pharmacology, 153, S230–S240. 10.1038/sj.bjp.0707491 17994114 PMC2268065

[phy215099-bib-0016] Kawabata, A. , Oono, Y. , Yonezawa, D. , Hiramatsu, K. , Inoi, N. , Sekiguchi, F. , Honjo, M. , Hirofuchi, M. , Kanke, T. , & Ishiwata, H. (2005). 2‐Furoyl‐LIGRL‐NH2, a potent agonist for proteinase‐activated receptor‐2, as a gastric mucosal cytoprotective agent in mice. British Journal of Pharmacology, 144(2), 212–219. 15655521 10.1038/sj.bjp.0706059 PMC1575994

[phy215099-bib-0017] Koh, S. D. , Ward, S. M. , & Sanders, K. M. (2012). Ionic conductances regulating the excitability of colonic smooth muscles. Neurogastroenterology and Motility, 24, 705–718. 10.1111/j.1365-2982.2012.01956.x 22726670 PMC4405144

[phy215099-bib-0018] Lee, S. , & Dong, H. H. (2017). FoxO integration of insulin signaling with glucose and lipid metabolism. Journal of Endocrinology, 233(2), R67–R79. 10.1530/JOE-17-0002 PMC5480241 28213398

[phy215099-bib-0019] Lembo, A. , & Camilleri, M. (2003). Chronic constipation. New England Journal of Medicine, 349, 1360–1368. 10.1056/NEJMra020995 14523145

[phy215099-bib-0020] Lu, H. , Zhang, C. , Song, N. , Lu, C. , Tong, L. , Huang, X. U. , Kim, Y.‐C. , Chen, J. , & Xu, W. (2017). Colonic PDGFRα overexpression accompanied forkhead transcription factor FOXO3 up‐regulation in STZ‐induced diabetic mice. Cellular Physiology and Biochemistry, 43, 158–171. 10.1159/000480335 28848093

[phy215099-bib-0021] McGuire, J. J. , Saifeddine, M. , Triggle, C. R. , Sun, K. , & Hollenberg, M. D. (2004). 2‐furoyl‐LIGRLO‐amide: a potent and selective proteinase‐activated receptor 2 agonist. Journal of Pharmacology and Experimental Therapeutics, 309(3), 1124–1131. 10.1124/jpet.103.064584 14976230

[phy215099-bib-0022] Ordog, T. (2008). Interstitial cells of Cajal in diabetic gastroenteropathy. Neurogastroenterology and Motility, 20, 8–18. 10.1111/j.1365-2982.2007.01056.x 18173559

[phy215099-bib-0023] Sanders, K. M. , Koh, S. D. , Ro, S. , & Ward, S. M. (2012). Regulation of gastrointestinal motility–insights from smooth muscle biology. Nature Reviews Gastroenterology & Hepatology, 9, 633–645. 22965426 10.1038/nrgastro.2012.168 PMC4793911

[phy215099-bib-0024] Sanders, K. M. , Zhu, M. H. , Britton, F. , Koh, S. D. , & Ward, S. M. (2012). Anoctamins and gastrointestinal smooth muscle excitability. Experimental Physiology, 97(2), 200–206. 10.1113/expphysiol.2011.058248 22002868 PMC3272164

[phy215099-bib-0025] Sato, K. , Ninomiya, H. , Ohkura, S. , Ozaki, H. , & Nasu, T. (2006). Impairment of PAR‐2‐mediated relaxation system in colonic smooth muscle after intestinal inflammation. British Journal of Pharmacology, 148, 200–207. 10.1038/sj.bjp.0706717 16520739 PMC1617061

[phy215099-bib-0026] Song, N. N. , Lu, H. L. , Lu, C. , Tong, L. , Huang, S. Q. , Huang, X. , Chen, J. , Kim, Y. C. , & Xu, W. X. (2018). Diabetes‐induced colonic slow transit mediated by the up‐regulation of PDGFRα+ cells/SK3 in streptozotocin‐induced diabetic mice. Neurogastroenterology and Motility, 30, e13326. 10.1111/nmo.13326 29521017

[phy215099-bib-0027] Sung, T. S. , Kim, H. U. , Kim, J. H. , Lu, H. , Sanders, K. M. , & Koh, S. D. (2015). Protease‐activated receptors modulate excitability of murine colonic smooth muscles by differential effects on interstitial cells. Journal of Physiology, 593, 1169–1181. 10.1113/jphysiol.2014.285148 PMC4358678 25641660

[phy215099-bib-0028] Talley, N. J. , Young, L. , Bytzer, P. , Hammer, J. , Leemon, M. , Jones, M. , & Horowitz, M. (2001). Impact of chronic gastrointestinal symptoms in diabetes mellitus on health‐related quality of life. Am J Gastroenterology, 96, 71–75. 10.1111/j.1572-0241.2001.03350.x 11197290

[phy215099-bib-0029] Vergnolle, N. (2005). Clinical relevance of proteinase activated receptors (PARS) in the gut. Gut, 54, 867–874. 10.1136/gut.2004.048876 15888798 PMC1774539

[phy215099-bib-0030] Wei, M. , Liu, Y. , Zheng, M. , Wang, L. , Ma, F. , Qi, Y. , & Liu, G. (2019). Upregulation of protease‐activated receptor 2 promotes proliferation and migration of human vascular smooth muscle cells (VSMCs). Medical Science Monitor, 25, 8854–8862. 10.12659/MSM.917865 31756174 PMC6883764

[phy215099-bib-0031] Yuan, J. P. , Wang, L. W. , Qu, A. P. , Chen, J. M. , Xiang, Q. M. , Chen, C. , Sun, S.‐R. , Pang, D.‐W. , Liu, J. , & Li, Y. (2015). Quantum dots‐based quantitative and in situ multiple imaging on ki67 and cytokeratin to improve ki67 assessment in breast cancer. PLoS One, 10, e0122734. 10.1371/journal.pone.0122734 25856425 PMC4391934

[phy215099-bib-0032] Zhao, P. , Metcalf, M. , & Bunnett, N. W. (2014). Biased signaling of protease‐activated receptors. Frontiers in Endocrinology, 5, 67. 10.3389/fendo.2014.00067 24860547 PMC4026716

[phy215099-bib-0033] Zhu, T. , Sennlaub, F. , Beauchamp, M. H. , Fan, L. , Joyal, J. S. , Checchin, D. , Nim, S. , Lachapelle, P. , Sirinyan, M. , Hou, X. , & Bossolasco, M. (2006). Proangiogenic effects of protease‐activated receptor 2 are tumor necrosis factor‐alpha and consecutively Tie2 dependent. Arteriosclerosis, Thrombosis, and Vascular Biology, 26(4), 744–750. 10.1161/01.ATV.0000205591.88522.d4 16439712

